# Intra-Couple Wealth Inequality: What’s Socio-Demographics Got to Do with it?

**DOI:** 10.1007/s10680-022-09633-4

**Published:** 2022-08-03

**Authors:** Miriam Rehm, Alyssa Schneebaum, Barbara Schuster

**Affiliations:** 1 Institute of Socio-Economics, University of Duisburg-Essen, Lotharstrasse 20-22, 47057 Duisburg, Germany; 2 Department of Economics, Vienna University of Economics and Business, Welthandelsplatz 1, 1020 Vienna, Austria; 3 The New School for Social Research, 66 West 12th Street, New York, NY 10011 USA

**Keywords:** Wealth, Gender wealth gap, Demographics, Bargaining power

## Abstract

Existing literature shows that on average and across countries, men have higher levels of wealth than women. However, very little is known about the gender-specific wealth gap *within couples*. This paper studies this phenomenon for the first time in Austria. The particular focus of the paper is on the relationship between the socio-demographic characteristics of the couple and the couple’s gender wealth gap. We focus on how age, education, marital status, fertility, migratory background, and the gender of the respondent are related to the wealth gap within a couple. In both bivariate and multivariate analyses, we find evidence in support of the hypothesis that bargaining power plays an important role in the intra-couple gender wealth gap in Austria. Immigrant women living in a couple with native men, and, among natives, couples in which the man is much older on average, have larger gender wealth gaps. Furthermore, couples in which the woman is the “financially most knowledgeable person” in the household have consistently lower gender wealth gaps.

## Gender and Wealth

Research on wealth inequality has boomed in the last decade, substantially improving our understanding of how wealth is distributed across households. With the development of this literature, social scientists have also gained new insights into the gender dimensions of wealth inequality. Despite our growing understanding of wealth inequality *across* households, we have little empirical knowledge about wealth inequality *within* households. Like income, individual wealth holdings are likely positively correlated with bargaining and decision-making power. The study of wealth gaps within couples thus allows us to get closer to understanding power differences within couples and households, hitherto often neglected since standard economic models did not include the decision-making process within the household. They assumed that resources within a household are distributed according to need by using adult equivalents, but disregarding differences in power (Haddad & Kanbur, 1990). From the point of view of measuring aggregate wealth inequality, measures of wealth inequality that treat the household as a single unit with intra-couple economic equality underestimate “true” inequality, unless wealth is distributed perfectly equally between partners within the household (Kanbur, 2018; De Vreyer & Lambert, 2020). However, several researchers claim that the assumption of equal sharing within a household is incomplete and misleading and emphasize instead the importance of an intra-household analysis of resource allocation (Chiappori & Meghir, 2015; Bütikofer & Gerfin, 2017; Sauer et al., 2021). This paper follows this approach and uses unique data on the intra-household distribution of wealth ownership in Austria to study gendered wealth inequality within couples, and how this is mediated by bargaining power.

Most empirical studies looking at the gender wealth gap assess the difference in net wealth of households headed by men versus women, and find that men have higher wealth holdings than women in both the raw data and in multivariate analysis (Deere & Doss, 2006; Chang, 2010; Ruel & Hauser, 2013; Schneebaum et al., 2018). There is also a significant minority of studies that find no gender-specific wealth gap. This is the case for the subgroup of young households in Schmidt and Sevak (2006) and for the marriage wealth premium in Lersch (2017) in the full models in these analyses. Despite the contributions of these studies, the intra-household distribution of wealth—that is, the way in which wealth is distributed within a household—has largely remained a black box. Much of the reason why is because almost all existing datasets collect information on wealth at the household, not person, level.

This paper addresses this gap in the literature. We use data from the second wave (data collected in 2014–2015) of the Household Finance and Consumption Survey (HFCS), which has been a major contributor to the boom in analyses of the distribution of wealth in Europe (Household Finance and Consumption Network (HFCN), 2019). These are the first data to make it possible to investigate the socio-demographic determinants of the gender wealth gap at the personal level in Austria.

Austria is one of just a handful of high-income countries for which nationally representative person-level wealth data are available. For Germany, Sierminska et al. (2010), Grabka et al. (2015), Lersch (2017) and Sierminska et al. (2018) have done extensive research using the Socio-Economic Panel (SOEP) and for France, the French HFCS has been analyzed by Frémeaux and Leturcq (2020). The British Household Panel Survey and the British Wealth and Assets Survey also contain individual-level wealth data. Moreover, the Household, Income and Labour Dynamics in Australia (HILDA) survey panel data contain person-level wealth data on bank accounts, superannuation, debt, and credit cards, and the data from the Survey of Income and Program Participation (SIPP) in the USA contain information on assets and debts for all people in a household, including whether these assets and debt were owned individually or jointly.^1^

Austria is an especially interesting case to study intra-couple gender wealth gaps, because the distribution of its household wealth is highly unequal in international comparison, and the question remains open whether intra-household dynamics play a role in this. Concretely, Austria’s mean-median ratio, which indicates the right-skewedness of the wealth distribution, is fifth highest among OECD countries (Balestra & Tonkin, 2018), and the Gini coefficient amounts to 0.73 in the 2014 HFCS data (fourth highest in the Euro area). This high level of inequality is linked to at least two major institutional issues. Austria has a large share of renters (Fessler et al., 2016; Pfeffer & Waitkus, 2021b). Relatively few households own their homes, but those that do have much higher wealth than renters. At the same time, social services crowd out a relatively larger share of private wealth—especially with regard to social housing—in Austria, making individual wealth accumulation less important for economic well-being than it is in countries with fewer social services. Given both of these conditions, inheritances play a major role in determining wealth in Austria (Piketty, 2014; Fessler & Schürz, 2018).

In studying the wealth gap within households, the unique contribution of this paper is its focus on the ways in which couples’ socio-demographic characteristics—their age, education, marital status, fertility, and migratory background—relate to their gender-specific distribution of wealth, and in particular whether this can be explained by differences in bargaining power. Concretely, we assess (both theoretically and empirically) the ways in which these five socio-demographic characteristics may be related to the unequal distribution of wealth within couples, and whether this wealth distribution can be the result of unequal leverage in bargaining between the man and the woman in the couple. In this regard, we especially focus on the question whether the gender of the respondent is correlated with the gender wealth gap, which we hypothesize to be the case since the respondent is by definition the financially most knowledgeable person who may also invest their wealth strategically. Our variables to help explain the couple-level gender wealth gap are also at the level of the couple: the couple’s age difference, the composition of their countries of origin, and the highest level of education in the couple are examples. By structuring the analysis in this way, we can assess how these characteristics relate (or do not relate) to intra-couple wealth inequality.

Empirically, we present the relationship between the couple-level socio-demographic characteristics and the intra-couple gender wealth gap in both bivariate and multivariate analyses. The latter employs OLS analyses to assess the correlation between the socio-demographics and the mean wealth gap while controlling for other socio-demographic and economic characteristics; quantile regressions predicting the median wealth gap and the wealth gap at the 25th and 75th percentiles are found in the appendix. The main findings of the paper show that indeed, some of the socio-demographic variables studied here are strongly related to intra-couple wealth gaps. The difference in the ages of the two members of the couple is a particularly powerful characteristic related to the gender wealth gap. Moreover, couples in which the man is native-born and the woman is an immigrant have a large gender wealth gap. The gender of the respondent is also consistently related to the gender wealth gap. All of these variables may have a direct influence on the gender wealth gap, and they are consistent with a bargaining power interpretation of the gender wealth gap.

## Literature and Theory

### Literature on the Gender Wealth Gap

Although the theoretical literature emphasizes that household resources (both income and wealth) cannot be assumed to be pooled and shared equally (Ponthieux & Meurs, 2015), the fact that wealth data are typically collected at the household level has limited the number of studies that assess the wealth gap between men and women within the household. Many existing studies have therefore been restricted to analyzing wealth differentials by gender by comparing the wealth holdings of single-adult households (Schmidt & Sevak, 2006; Schneebaum et al., 2018); by defining the household through a representative member (Ruel & Hauser, 2013); or by assessing the gender wealth gap in particular components of wealth sometimes reported at the individual level, such as pensions (Neelakantan & Chang, 2010). The important exceptions to the literature’s reliance on household-level data are based on the wealth module of the German Socio-economic Panel (Sierminska et al., 2010; Grabka et al., 2015; Sierminska et al., 2018), and the French Household Finance and Consumption Survey (HFCS) and its national precursor, the Life History and Wealth Survey (Frémeaux & Leturcq, 2020). In addition to Germany and France, there are seminal papers investigating the gender wealth gap in the USA (Schmidt & Sevak, 2006; Ruel & Hauser, 2013; Yamokoski & Keister, 2006). Finally, Schneebaum et al. (2018) analyze the gap in several European countries.

Most studies of the gender wealth gap include some reference to (socio-)demographic characteristics; however, the focus of the analysis often lies on other factors, such as labor market characteristics, so the information on socio-demographic characteristics functions largely as control variables (Schmidt & Sevak, 2006; Neelakantan & Chang, 2010; Grabka et al., 2015; Ruel & Hauser, 2013). The main exception is Yamokoski and Keister (2006), who investigate the effect of education, marriage, and fertility on the gender wealth gap, but do not include immigration as a socio-demographic characteristic. Other contributions focus on education (Sierminska et al., 2010), marital status (Sierminska et al., 2018; Frémeaux & Leturcq, 2020), or immigration (Bauer et al., 2011).

This paper expands the literature on the gender wealth gap by providing evidence of the determinants of intra-household gender wealth differences in Austria based on the HFCS, looking especially at the role of socio-demographic characteristics on the gap. The rest of this section discusses the institutions framing the relevance of these socio-demographic characteristics, and develops hypotheses as to the direction of their effect. It is worthwhile to mention the limitations of our study upfront. Our data do not contain information on asset categories at the individual level; therefore, we can neither examine intra-household wealth gaps in real or financial assets, nor put a focus on housing wealth, but instead can study only gaps in net wealth. We hope for better data availability in the future, since Pfeffer and Waitkus (2021b) highlight the importance of housing equity in the analysis of wealth inequality and D’Alessio (2018) shows with a reconstruction of individual wealth data in Italy that the gender wealth gap is larger for financial assets than for real assets. For now, we can only draw on Schneebaum et al. (2018), who investigate the distribution of asset categories among European single-adult households and find a gender wealth gap of 44% in gross wealth, 64% in real wealth, 50% in financial wealth and 33% in the value of the main residence of single households at the top of the distribution in Austria.

### Bargaining Power

Intra-household gender relations are often characterized by differences in decision-making and bargaining power between household members, particularly power differences between women and men. The bargaining power framework reveals how gender asymmetries are constructed and contested (Agarwal, 1997). Bargaining power is intimately related with all socio-demographic characteristics in our analysis. In line with the hypothesis that greater economic resources result in greater bargaining power, the most common approach is to proxy bargaining power with earnings or an individual’s contribution to total household income (Friedberg & Webb, 2006; Bütikofer et al., 2009; Burger & Kreuser, 2017). Other labor market characteristics, such as employment, are also correlated with higher involvement in the decision-making process and thus more bargaining power (Antman, 2014).

Bargaining power measures beyond labor market features have been studied as well. A spouse’s productivity in household production might be a source of bargaining power (Pollak, 2005), higher levels of education impact earnings and thus positively affect bargaining power (Datta Gupta & Stratton, 2010), but also physical attractiveness in combination with age can make a significant difference in power relations (Esping-Andersen & Schmitt, 2019). The gaps in education and age between spouses are also related to the distribution of bargaining power within a couple. Education is an important source of women’s outside marriage options and thus increases her intra-household bargaining power. The age gap is linked to the outside options on the marriage market. On the one hand, a large intra-couple age gap is likely to increase younger wives’ bargaining power, while on the other hand, older wives benefit from more life experience that equips them with greater influence on decision making (Afoakwah et al., 2020).

The literature has found that ultimately, the last word on financial decision making, and therefore bargaining power, is strongly related to the magnitude of the wealth gap. When the couple claims that the male partner makes the decisions, the intra-couple wealth gap is greatest and those couples are also among the wealthiest households. However, if the woman has the final say, the intra-couple wealth gap is the lowest, but such couples also have the lowest average net wealth (Grabka et al., 2015). We therefore expect that higher bargaining power of women is related to lower intra-household wealth gaps.

Along with measures of our socio-demographic characteristics, which are related to bargaining power, our data allow us a unique proxy of bargaining power via the gender of the survey respondent. By definition, the survey respondent is the “financially most knowledgeable person” in the household, and we suppose that this person has more bargaining power than their partner because of this financial knowledge. If the woman responds, this suggests that she is involved in financial decision making within the household and consequently has more bargaining power than a woman in a household where the man is the financially most knowledgeable person and survey respondent. High gender gaps in employment, wages, and working hours leave women in Austria with relatively less income and thus less financial capital to build up wealth. Therefore, we would expect a financially knowledgeable woman to invest her wealth strategically to accumulate above-average wealth levels and to narrow the gap to her male partner, who starts from a better position in the accumulation process.

Besides bargaining power, differences in reporting might also influence the results depending on the gender of the survey respondent. Some studies have found evidence that husbands tend to over-report while wives rather under-report their own income (Singh et al., 2010), and that husbands report more income and gross assets than their wives, whereas wives report higher levels of debt than their husbands (Zagorsky, 2003). Time availability might also play a role in survey response, but this is not obvious because even if one person is more likely to be available to respond to the survey, they will not actually do so if they do not know the answer to the questions. Moreover, Lindamood and Hanna (2006) explored response behavior to the Survey of Consumer Finances (SCF) for older couple households and found that if a person is not employed and therefore has more free time than their partner, they are not more likely to be the respondent.

### Age

Wealth inequality within couples by age may be due to life-cycle factors (i.e., age) or cohort effects (Pfeffer & Waitkus, 2021a).^2^ The life-cycle hypothesis predicts that resources are accumulated during the economically active years and are spent down in retirement (Modigliani, 1966). Since men are typically older than women in couples and have thus had more time to accumulate wealth, we would expect a positive correlation between the *age difference* within the couple and the gender wealth gap. Higher age may be a proxy for higher bargaining power of one partner within the couple (Esping-Andersen & Schmitt, 2019; Afoakwah et al., 2020). We thus look at the *age difference* of the people within the couple to capture the role of age differences on the intra-couple wealth gap.

Furthermore, cohort effects for intra-couple wealth inequality refer to differences in wealth accumulation behavior between different age groups. Social and cultural norms change over time, which may lead to a more (or less) equal sharing of wealth within the couple, and thus a direct variation of the gender wealth gap within older couples relative to younger couples. It is thus important to capture not just the role of the age difference among members of a couple, but also the role of the age cohort in which a couple is. While untangling age from cohort effects is empirically difficult, we attempt to do so by looking at the *average age* of the couple as an indicator of cohort effects^3^ on the intra-couple gender wealth gap on the one hand and *age gaps* in the couple to capture bargaining power on the other hand.

Finally, cohort effects may be linked to other variables in our analysis. For instance, legal institutions around property sharing in marriage have changed over time (Floßmann, 2006). In our empirical analysis, we interact age with marital status to investigate this effect. Women’s educational attainment has also changed across cohorts (Fessler & Schneebaum, 2012). In addition, social norms around women’s labor force participation have changed, lowering earning differences in the couple over time. The latter two examples show the importance of controlling for cohort effects via the average age variable (though sample size constraints make interactions unreasonable).

### Education

Like age, the level of education is typically positively correlated with wealth (Pfeffer, 2018). Possible channels may either be the link of education to work income, or between education and financial literacy, and thus higher capital income (Cupák et al., 2018). At the couple level, if there is a *difference in education* that favors men, then this would be another possible explanation of the gender wealth gap. Moreover, such a gap in education between partners and the gender wealth gap might also indicate an imbalance in bargaining power within the couple, which could translate into a wealth gap.

Assortative mating—the preference for partners with the same or similar level of education^4^ (Greenwood et al., 2014; De Hauw et al., 2017; Boertien & Permanyer, 2019)—has an opposite effect on wealth inequality between households and within households: on the one hand, it raises wealth inequality overall (for income, see, e.g., Eika et al. (2019)), since random partner choice would more often match high wealth individuals to low wealth individuals, which then leads to lower average wealth inequality at the household level. On the other hand, educational homogamy should, if anything, reduce the intra-couple gender wealth gap. The reason is that assortative mating suggests that both partners have similar capacities for wealth accumulation, so that there are presumably lower returns to specialization on market versus non-market work. In addition, if partners are similar in terms of education, then there will also be less of an imbalance in bargaining power (Datta Gupta & Stratton, 2010). This in turn may lead to more similar wealth levels of the partners.

Finally, women’s educational attainment in most developed countries has caught up to that of men, and in Austria, young women are now more likely than young men to complete tertiary degrees (OECD, 2021). Such changes in educational attainment across cohorts, including that the development has quite gender-specific dynamics (Hek et al., 2016), are likely to impact the intra-couple gender wealth gap. Though it would be difficult to tease out these effects empirically because of small sample sizes, the role of gender-specific changes to educational attainment across cohorts should be captured in our econometric models via the average age variable.

### Marital Status

The empirical literature consistently documents a marriage wealth premium relative to single people or cohabiting couples (Keister, 2003; Sierminska et al., 2010; Vespa & Painter, 2011; Addo & Lichter, 2013; Painter et al., 2015; Lersch, 2017; Kapelle & Lersch, 2020). Apart from economies of scale, this may be due to a longer planning horizon and increased trust due to the higher commitment level of married couples, which may in turn increase specialization, total work hours of the couple, or investment (Lersch, 2017; Sierminska et al., 2018). While the *level* of wealth thus rises with marriage, it is much less clear whether marriage is also linked to a higher *difference* in the wealth levels of partners; that is, whether the gender wealth gap differs by marital status. There is evidence that spouses who first lived together and only later got married are more likely to opt for separation of property than spouses who married directly. However, the selection effects that drive some couples into premarital cohabitation overlap with those of separation of property, which diminishes the effect of premarital cohabitation on the choice of the property regime (Vitali & Fraboni, 2022).

The theoretical expectations for the effect of marital status on the gender wealth gap are not clear *a priori*. On the one hand, increased commitment arising from the sociocultural institution of the marriage pact may lead to more equality in the intra-marital distribution of assets. On the other hand, increased specialization may lead to a weaker labor market attachment of the partner specializing in the household and child care, which may raise the gender wealth gap. Finally, legal questions surrounding asset ownership in marriage might play an enhancing or diminishing role for the gender wealth gap, in particular community versus separate wealth ownership of couples (see also (Frémeaux & Leturcq, 2022)).

Regarding the legal institutions, the General Civil Code of Austria of 1811^5^ defined the wife as legally subordinate to the husband with regard to wealth management (Floßmann, 2006), although the separation of property was the standard case. It assumed by default that the wife entrusted the husband with managing the wealth that she had brought into the marriage. The wife was further obliged to aid the husband in his gainful employment, but the husband retained management and use rights of assets acquired during the marriage (Lehner, 1987). The literature thus considers the Austrian legal system until the 1970s as “presumed administrative community” and “disguised communal property” ((Floßmann, 2008), p. 95).

The family law reform of 1978 strengthened the separate wealth ownership system, making the wife fully self-determined in owning and managing her wealth (Floßmann, 2006). The current Austrian property ownership system is thus the separation of property. In the event of divorce, the marital assets and savings that served the use of both spouses and that were accumulated during the marriage are divided evenly. Excluded from the division are items that were brought into the marriage, inherited, or gifted by third parties; as well as items for personal use or professional practice, and items related to a company. In case of the death of a partner, the spouse of the deceased has a statutory right of inheritance, with the amount of the inheritance being at least one-third, but also depending on which other relatives are entitled to inherit (Bundesministerium für Digitalisierung und Wirtschaftsstandort, 2022). Only a small share of couples deviates from the separation of property by signing a pre-nuptial marriage contract.^6^

One hypothesis that can be derived from these legal stipulations is that the gender wealth gap is ambivalently related to time of marriage, in Austria particularly those who likely married before 1978. On the one hand, a higher share of individualized wealth can lead to a higher gender wealth gap within couples if men own a larger share of individualized assets, as Frémeaux and Leturcq (2020) show. On the other hand, women were historically in a disadvantaged position with regard to wealth acquisition. The persistence of cultural norms formed by these legal institutions that had been in force for more than 150 years, however, might dilute the effect of the 1978 legal reforms.

### Fertility

Fertility may affect the wealth level of the couple through different channels. A couple’s wealth may either fall in response to higher fertility due to increased costs (and thus reduced savings), or rise due to a higher saving incentive (such as the need for larger housing, or precautionary saving for education). Fertility may also affect relative wealth, and thus the gender wealth gap. First, childbirth impacts relative earnings within the couple; work interruptions for child-rearing in particular correspond with large income gaps by gender (Kleven et al., 2019). Second, the relative wealth of partners may be affected positively by the presence of children (Grinstein-Weiss et al., 2008; Maroto, 2018) since they—like marriage—may increase commitment, and thus lead to enhanced wealth sharing and consequently a smaller gender wealth gap. Third, on the other hand, increased specialization on child care by one partner may atrophy both their financial literacy and their knowledge of the family’s financial affairs, which may be conducive to a larger gender wealth gap. For empirical purposes, it is important to disentangle the effects of age versus children (respectively, childlessness) on the intra-couple gender wealth gap, since the two have distinct effects.

### Immigration

Immigration policies affect the selection of immigrants by gender, as well as by their wealth ownership and their characteristics that determine their ability to acquire wealth. These policies may thus lead to a larger gender wealth gap if, for instance, immigrants are predominantly female and from low-wealth countries. Alternatively, immigrants may be selected from low-wealth groups within their countries, or immigration may be linked to the loss of property or inheritance claims in the country of origin. Furthermore, immigration might be linked to lower earnings due to less training and skills for the labor market in the destination country, and more limited information regarding financial investment opportunities. On the other hand, selection effects may also play a role in defining the remaining stock of immigrants in a country, when return (and repeat) migration is taken into account (Gobillon & Solignac, 2019).

In Austria, like in Germany and Switzerland, immigration during the labor shortages of the 1950s and especially 1960s was marked by temporary guest-worker policies (Hansen, 2003). In the early 1970s, however, Austria—like most European countries—reduced work immigration, and moved toward family reunification. Refugee immigration—which played some role in Austria in earlier decades (such as when the failed Hungarian uprising led to Hungarian refugees fleeing to Austria in 1956)—became especially salient after 1989, when opened borders, falling travel costs, and violent conflicts like the war in former Yugoslavia led to new immigration pressure.

Empirically, there is a wealth gap between immigrants and natives in Austria (Muckenhuber et al., 2021). This immigrant wealth gap manifests itself especially in the upper half of the distribution, where home and business ownership is particularly salient. Furthermore, there is evidence for catch-up or a cohort effect due to the immigration policies described above—the immigrant wealth gap for first generation immigrants is substantial, while second generation immigrants (very roughly speaking, the children of guest workers) are very similar to natives both in terms of their wealth and their socio-demographic characteristics (Muckenhuber et al., 2021). The main explanatory factors for the immigrant wealth gap at the top of the unconditional wealth distribution are inheritances and marital status; and age, children, education, and income for first generation immigrants (Muckenhuber et al., 2021).

Since the literature thus suggests that individuals with a *migratory background* have lower wealth on average, an immigrant wealth gap will only translate into a gender wealth gap if one of the partners in the couple has migratory background—not if both or neither do. Mixed immigrant-native couples in which the woman has a migratory background would thus have a larger gender wealth gap; the gender wealth gap should be smaller in couples where both partners are natives or both are immigrants; and it should be smallest in couples in which only the man has a migratory background.

## Data and Research Design

### Data: HFCS

We use data from the second wave of the Austrian Household Finance and Consumption Survey (HFCS), a dataset containing information on the real and financial assets, liabilities, and consumption of private households (Household Finance and Consumption Network (HFCN), 2017). The HFCS is coordinated by the European Central Bank (ECB) in close cooperation with the national central banks of the Eurosystem; in Austria, it is conducted by the Austrian National Bank (OeNB). Its second wave surveyed 2997 households in 2014 and 2015. The key data used in this study come from the so-called “non-core” data, which provide information on net wealth at the individual level. The only other country participating in the HFCS to contain person-level data is France; the gendered distribution of wealth found in those data are analyzed by Frémeaux and Leturcq (2020) and Frémeaux and Leturcq (2022).

In this paper, we analyze the distribution of wealth within (heterosexual) cohabiting couples, both married and unmarried. In the total dataset, there are 2,997 households. Our sample consists of the 1503 households in which there is a couple, that is, the households in which the survey respondent (the so-called “financially most knowledgeable person” in the household) indicates that another person in the same household is their spouse or partner. The men in our sample range in age from 20 to 85 (mean 54 and median 53) and the women range in age from 18–85 (mean and median 51). These individuals are older than the country average overall, because couple households account for just 52% of all Austrian households and there is an age-specific selection into cohabitation. The couples may be legally married or “simply” cohabiting; in the latter case, the individuals in the couple may be divorced or widowed from previous relationships or may be legally single (people who were divorced or widowed and later remarried would appear in the “married” group, as these categories refer to status at the time of the survey). In all parts of the analysis, we survey weight the data. Moreover, all computations are completed taking advantage of the multiple implicate structure of the data. There is no oversampling of wealthy households in the Austrian HFCS, but households in cities are oversampled.

### Variables

#### Outcome: Gender differences in Net wealth

Our key outcome variable is the gap in net wealth held by the male and female partners in a couple. Net wealth is defined as gross wealth minus total liabilities; gross wealth includes real and financial assets, while liabilities consist of collateralized and unsecured debt. In the HFCS, real assets comprise the main residence, other real estate property, vehicles, other valuables, and self-employment businesses. Financial assets are made up of deposits, mutual funds, bonds, shares, managed accounts, non-self-employment businesses, money owed to the household, other financial assets, and voluntary pension and life insurance plans. Collateralized debt includes mortgages on the main residence or on other real estate property; unsecured debt consists of overdrafts, credit card debt, and other unsecured loans. These wealth components are collected at the household level. Ownership shares of the household’s net wealth for each household member are provided by the respondent, who completes the survey for the whole household. We calculate the intra-household wealth gap as the difference in the net wealth held by the man and the woman in the couple. An interesting starting point is to note that about three quarters of couples report no gender wealth gap, as shown in Table 9. In multivariate analysis below, we focus some specifications on households with a gap only.

Table 1 shows the wealth level (in Euros) and the raw gender wealth gap (as a percent of the male’s wealth) among men and women in couples in Austria. The average couple household in Austria holds €356,553 in wealth; the median couple household has €173,683. On average, women hold about €150,000 compared to men’s €207,000, leading to a gap of about €58,400, or about 28% of the men’s average wealth—even including households who report no gender wealth gap in the analysis. At the median, the gap is about €13,900, or 17% of men’s wealth. The difference between the mean and the median in the wealth level of couples indicates that the data are highly right-skewed.

**Table 1:** Wealth holdings and the gender wealth gap

	Mean wealth	Gender gap		Median wealth	Gender gap	
		Absolute	Relative		Absolute	Relative
		(in €)	(as %)		(in €)	(as %)
Couples’ total wealth	356,553			173,683		
Female’s share	149,068	58,417	28	68,422	13,862	17
Male’s share	207,485			82,285		

The absolute gap is the Euro value of the difference in male versus female wealth. The relative gender wealth gap is the absolute difference in male versus female wealth relative to male wealth. Authors’ calculations on 2014 HFCS data

**Fig. 1:**
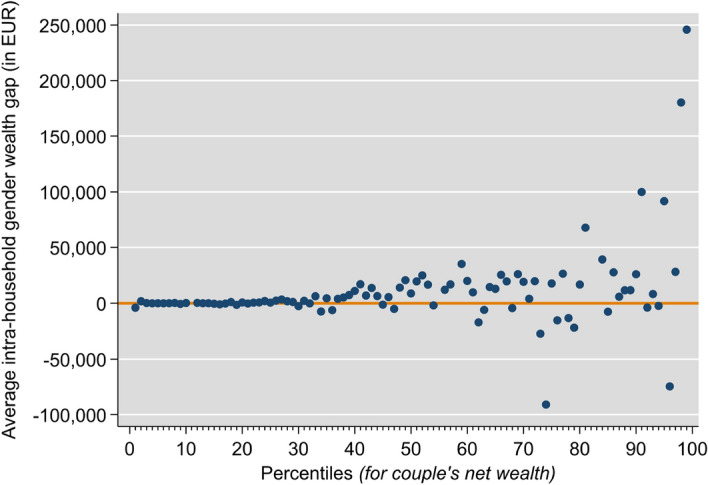
The raw gender wealth gap between women and men in couple households. *Notes:* Weights and multiple imputations taken into account. No values for percentiles 11, 55, 58, 82, 83 due to varying sets of implicates. Gender wealth gap is the difference between a man’s and woman’s net wealth. Authors’ calculations on 2014 HFCS data

**Fig. 2:**
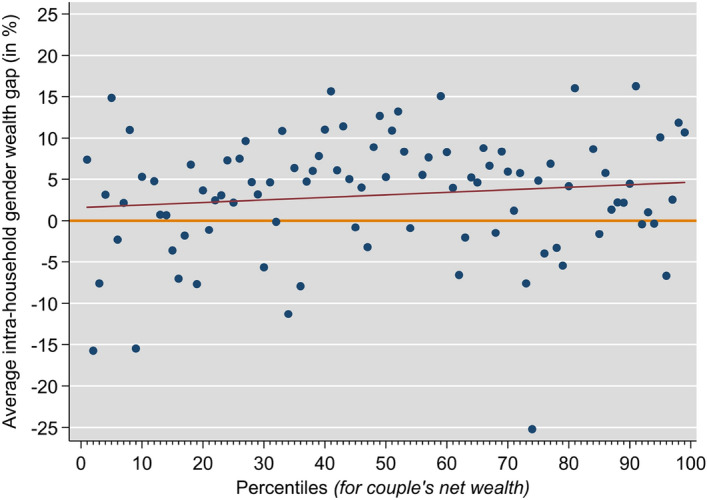
Gender wealth gap within couples by percentiles (in %). *Notes*: Weights and multiple imputations taken into account. No values for percentiles 11, 55, 58, 82, 83 due to varying sets of implicates. Gender wealth gap is defined as the difference between a man’s and woman’s net wealth compared to the couple’s total net wealth. Authors’ calculations on 2014 HFCS data

The data show that the average gender wealth gap in absolute terms rises across the unconditional distribution of couples’ net wealth percentiles. Figure 1 illustrates this distribution of the raw gender wealth gap, along the distribution of couples’ net wealth. The gap is generally higher the higher the level of wealth is. Further, as shown by Fig. 2 in the appendix, the gender wealth gap is also somewhat higher for wealthier households when the gap is measured as a percentage of household wealth.

#### Socio-Demographic Characteristics

In exploring the gender wealth gap, we look at five core explanatory socio-demographic variables that may relate to the intra-couple wealth gap. First is age, measured as the age gap within the couple (in categories of same age (the omitted category), less than five years, five to 10 years, and more than 10 years). We also control for the average age of the people in the couple to capture cohort effects. Second, we consider differences in the education level of the people in the couple. Education is measured in three categories (primary and lower secondary education—ISCED 0–2; upper secondary education—ISCED 3–4; and tertiary education—ISCED 5–6). The dummies here are the same education level (omitted category); and the man or the woman having either one or two categories higher education. Furthermore, we consider the role of the highest level of education among the two partners in the couple. Third, we measure migratory background, in which we define a person whose country of birth is other than Austria as an immigrant. At the household level, we consider whether the couple comprises both native Austrians, both immigrants, or just one immigrant, where the latter is specified by gender. Immigration is thus captured via four mutually exclusive dummy variables: only the woman is an immigrant; only the man is an immigrant; both partners are immigrants; or neither are immigrants (the omitted category). Fourth, we consider the marital status of the individuals in the couple by using a dummy variable indicating that the couple is currently married. While all couples in our sample are cohabiting, the omitted alternative to being married is that the members of the couple state that they are divorced, widowed, or legally unmarried, or any combination of these. We further include a dummy variable, indicating that the couple is married and “older”—that is, born before 1958—to account for the institutional changes around gender equality for married couples that occurred in 1978, as described in Sect. 2. Finally, we look at fertility, that is, the presence of children in the household, both in terms of the number of children present and the age of the youngest child in the household. A dummy variable thus indicates the number of children living in the household (none (the omitted category), one, or two), and another one captures the age of the youngest child (zero to five, or older).

Table 2 provides an overview of descriptive statistics; more detailed descriptives relating our socio-demographic characteristics to wealth and the gender wealth gap are below. Due to the small number of observations of divorced and widowed women and men, we do not control for these characteristics in the multivariate analysis in Sect. 5 below; we differentiate only between married and unmarried couples.

**Table 2:** Descriptive statistics for women and men in couple households

	Females	Males
Average age	50.9	53.6
Education: primary/lower secondary	20.1	10.7
Education: upper secondary	65.4	65.1
Education: tertiary	14.4	24.2
Immigrant	11.3	11.0
Married	92.8	92.8
Legally single	4.6	4.7
Divorced	2.0	2.2
Widowed	0.4	0.3
Share with children in household	30.3	
N observations	1503	1503

Authors’ calculations on 2014 HFCS data

In Sect. 4, we explore the relationship between these five socio-demographic variables of interest and the gender wealth gap for men and women in couple households. We use these bivariate analyses to get a sense of how these socio-demographic characteristics are related to wealth holdings for men and women to better understand how they will matter for the gender wealth gap within couples. For this part of the analysis, the outcome variable of interest is net wealth for men and women. In Sect. 5, we turn to a multivariate analysis of the socio-demographic determinants of the intra-household wealth gap. In other words, we shift our outcome variable of interest from the average or median net wealth of all women or all men in couple households to the gender wealth gap within individual households.

#### Survey Respondent: Financially Most Knowledgeable Person

Either the man or the woman in the couple can respond to the wealth survey. Table 3 shows wealth holdings and the raw gender wealth gap by gender of the respondent among men and women in couples. The man is the respondent in more than half of couples (803 couples, versus 700 couples with a female respondent).

Couples with a female respondent—those in which the woman likely has more bargaining power compared to couples whose male partners are the “financially most knowledgeable” member of the household —have considerably lower gender wealth gaps at the mean and the median compared to couples with a male respondent. In fact, there is no gap between the partners’ mean wealth holdings in couples with a female respondent; both partners have on average about €138,000. In couples in which the survey respondent was the man, however, the intra-couple wealth gap amounts to 41%, and men, on average, hold €110,000 more wealth than their partners. This pattern persists at the median: the median wealth gap in couples with a female respondent is only 2% and in favor of women, while the gap amounts to 27% in favor of men in couples with a male respondent. Furthermore, wealth levels are higher for both partners when the respondent is the man and lower in case of female respondents.

In addition (see Table 9 in the Appendix), female respondents are somewhat more likely to report no gap; and if they do, they tend to report a gap in their favor, while male respondents report an (often larger) gap in their favor. Despite small sample sizes in the comparison groups, the data on the gender of the respondent also indicate that married couples share their wealth more equally, which may indicate increased commitment. Other features, such as a higher gender wealth gap when children are present, may suggest increased specialization.

As discussed in Sect. 2, there are a number of possible explanations for this finding. On the one hand, the ECB requests the “financially most knowledgeable powerson” in the household to respond to the survey. If this is the reason for the choice of respondent, then this person is likely to have more bargaining power than their partner. Bargaining power, in turn, could explain the larger wealth gap when the man responds and smaller wealth gap when the woman responds. On the other hand, however, we cannot rule out cognitive bias, or gender differences in perceiving, or acting upon, social desirability.

**Table 3:** Wealth holdings and the gender wealth gap by gender of respondent

	Mean wealth	Gender gap		Median wealth	Gender gap	
		Absolute	Relative		Absolute	Relative
		(in €)	(as %)		(in €)	(as %)
*Male respondent*						
Male	270,307	110,995	41.1	99,347	27,085	27.3
Female	159,312			72,262		
*Female respondent*						
Male	138,830	957	0.7	63,825	− 1395	− 2.2
Female	137,873			65,220		

Gender wealth gap relative to male wealth. Authors’ calculations on 2014 HFCS data

### Empirical Approaches

In the next section, we present results of a bivariate analysis, in which we calculate and describe the level and gender gaps in wealth based on the socio-demographic characteristics of interest. Again, all means and medians in this section are computed using survey weights and employing the multiple imputation structure of the data.

We then approach the multivariate analysis of intra-couple wealth inequality via OLS in Sect. 5, that is, we predict the wealth gap within each couple based on the couple’s socio-demographic and other characteristics. Since the data are right-skewed and there are zero and negative values for net wealth in the data, we use the inverse hyperbolic sine (IHS) transformation of net wealth as the outcome variable.^7^

As discussed in Sect. 3.2.2 above, our socio-demographic control variables are age, education, immigration, marital status, and fertility. Apart from a dummy variable for the gender of the respondent, we include further controls in our model to predict the wealth gap within households: for both people, the employment status (employee, self-employed, employer, unemployed, not in labor force, or retired); the hours worked (full-time or part-time); labor market attachment (the number of years worked divided by potential work years, i.e., age minus 18); and a dummy variable indicating whether the household previously received an inheritance.

## Bivariate Results

This section presents the co-variation of the gender wealth gap with each of the socio-demographic characteristics of interest individually. Our goal is to show how the gender wealth gap varies by age, education, marital status, the presence and age of children, and migratory background. This analysis cannot isolate the relationship between any of these individual socio-demographic characteristics and the gender wealth gap, but it can give an initial picture of how they are correlated with the gender wealth gap. Table 4 shows the sample size, the share in the population, the mean and median wealth level of women and men in couples, and the relative gender gap at the mean and median for each of these variables.

**Table 4:** Wealth and wealth gaps by socio-demographic characteristics

	Sample	Share	Mean	Mean gap	Median	Median gap
*Same age*						
Women	132	8.6	190,777	− 0.2	107,941	6.5
Men			190,455		115,442	
*Woman is younger*						
Women	1,113	73.3	146,286	12.3	64,836	20.9
Men			166,785		81,921	
*Woman is older*						
Women	258	18.2	140,521	63.0	73,776	− 0.4
Men			379,721		73,513	
*Same education*						
Women	964	63.7	140,813	36.2	68,219	13.7
Men			220,740		79,069	
*Woman less educated*						
Women	406	27.1	161,030	11.1	61,990	28.0
Men			181,198		86,051	
*Woman more educated*						
Women	133	9.2	171,000	11.5	94,017	− 1.6
Men			193,320		92,551	
*Married*						
Women	1,399	92.8	154,034	28.8	73,661	15.6
Men			216,343		87,290	
*Unmarried*						
Women	104	7.2	84,792	8.7	21,010	30.0
Men			92,833		30,030	
*No children*						
Women	873	56.0	136,591	39.5	65,752	13.7
Men			225,720		76,186	
*Youngest child 0–5*						
Women	199	13.7	79,801	25.9	22,732	42.4
Men			107,751		39,475	
*Youngest child 6+*						
Women	431	30.2	203,594	7.0	108,144	9.8
Men			218,917		119,948	
*Neither immigrant*						
Women	1,253	84.2	152,013	30.9	84,364	12.8
Men			219,981		96,793	
*Both immigrant*						
Women	102	6.5	53,961	− 3.2	9770	− 9.3
Men			52,305		8,940	
*Only female immigrant*						
Women	75	4.8	102,255	23.3	51,106	40.7
Men			133,376		86,204	
*Only male immigrant*						
Women	73	4.5	280,321	− 1.4	46,152	29.0
Men			276,417		64,963	

Gender wealth gap relative to male wealth. Authors’ calculations on 2014 HFCS data. Survey weights and multiple imputation structure employed in all calculations

As discussed in Sect. 2, we expect the age difference of the two partners to correlate with the intra-couple gender wealth gap—the older partner (more often the man) will likely have more wealth. Table 4 shows that indeed, when the members of the couples are the same age, there is virtually no gap at the mean, and a small one at the median. When the woman is younger—which is the case in the majority of couples—there is a positive wealth gap at both the mean and median. When the woman is older, there is a negligible negative gap at the median, but a large one on average. However, the detailed Table 11 in the Appendix shows that the larger gap at the mean is due to couples with a small age difference— couples in which the woman is less than five years older than the man. Note too, that the very large gap in couples in which the woman is more than ten years older than the man should be treated with caution due to the limited number of observations.

While the age gap in couples is thus broadly positively correlated with the gender wealth gap in our data, more fine-grained analysis in Appendix Table 11 shows that this finding is not symmetric for both genders. Women need to be considerably older for the gender wealth gap to be in their favor. From the perspective of bargaining power, this suggests that women require more leverage through their (even) higher age in order to achieve equal wealth as their male partner.

**Fig. 3:**
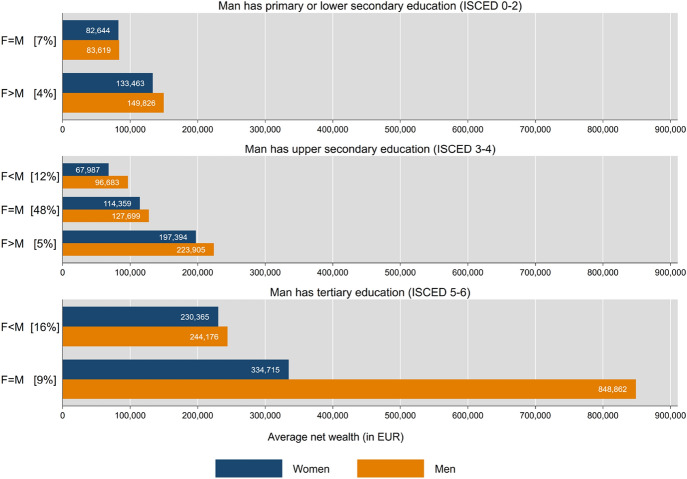
Comparison of women’s and men’s education levels in couples with regard to their mean net wealth. *Notes*: Weights and multiple imputations taken into account. Values in brackets refer to the shares of the respective couples. Authors’ calculations on 2014 HFCS data

**Fig. 4:**
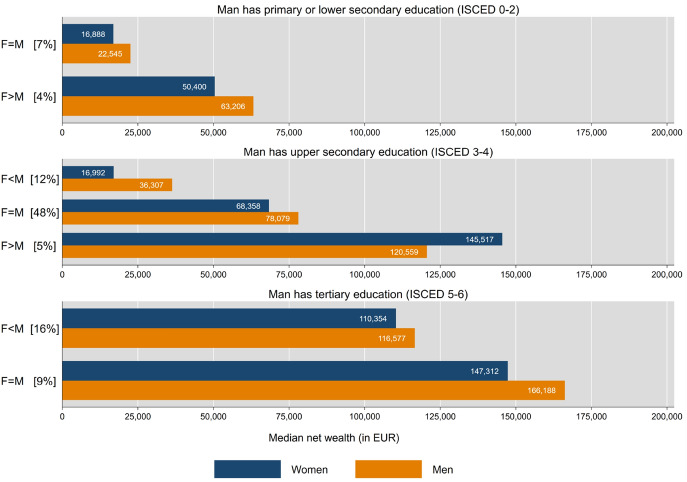
Comparison of women’s and men’s education levels in couples with regard to their median net wealth. *Notes*: Weights and multiple imputations taken into account. Values in brackets refer to the shares of the respective couples. Authors’ calculations on 2014 HFCS data

As with age, we expect the relative education level between members of a couple to contribute to a gender wealth gap within the couple. This is again only partly corroborated by the data (Table 4). At the median, the relationship is as expected: The gender wealth gap is larger when the woman in the couple is less educated than the man, compared to when both have the same level of formal education. The gap is negative (if small) when the woman is more educated. At the mean, the gap is positive irrespective of the woman’s education level relative to the man; it is largest when the woman and the man have the same level of education. The more detailed data by level of education in Figs. 3 and 4, Table 11 in the Appendix show that the woman’s wealth in couples is only higher in one of the 14 possible pairings.^8^ In sum, our data thus show that education does not close the gender wealth gap persistently in Austria. Even when women are more highly educated than men, a gender wealth gap persists on average. At the median, though, education does help: in the relatively few couples in which the woman is more highly educated, the median wealth gap is in her favor.

The description of the legal context in Sect. 2 indicated that we should expect a larger gender wealth gap among married couples, especially older ones. The bivariate evidence in Table 4 shows that there is a positive gender wealth gap both at the mean and at the median, for both married and unmarried couples. However, by comparing mean and median we see that this gender wealth gap is right-skewed in married couples, and left-skewed in unmarried couples. Moreover, the data confirm the marriage wealth premium found in the literature (that is, wealth levels are higher in married couples). In combination with the higher wealth gap in married couples, we see evidence that in couples with higher levels of wealth, the gender wealth gap tends to be larger (as in Fig. 1). This finding supports the hypotheses that women in married couples benefit from higher wealth accumulation through increased commitment, but that their weaker labor market attachment and/or bargaining power affects their relative wealth within the couple.

Theory provides arguments both for a higher and for a lower gender wealth gap due to children present in the household (see Sect. 2). The data show that the gender wealth gap is large and right-skewed in the group of couples without children. When there are children present, the gap falls (both on average and at the median), also with the age of the children. More fine-grained analysis in Table 11 shows that the gap is largest when there are two children present in the household (as opposed to one child or three or more children). Viewed in conjunction, this evidence provides some support for the hypothesis that more attachment leads to more shared wealth: the more children a couple has, and the longer the union lasts with children (as indicated by the age of the children), the lower the wealth gap within the couple. Yet, women’s labor market attachment may well play a role in explaining the larger gender wealth gaps when children are young, and when there are two children present.

Finally, as discussed in Sect. 2, we hypothesize that immigrants will have lower wealth than their native partners; and that the gender wealth gap is smaller in couples in which both people have the same immigration status (immigrant or native). Table 4 shows that the average gender wealth gap is, in fact, largest in the majority population—couples in which both partners are natives—and it is right-skewed. Couples comprised of two immigrants have much lower net wealth than natives, and their gender wealth gap is in favor of women both at the median and the mean. In couples where only the woman has a migratory background, the gender wealth gap re-emerges, and it is large both at the mean and the median. If only the man in the couple has a migratory background, then the gender wealth gap on average disappears, although it remains large at the median. The migratory background may thus work in mixed couples as hypothesized; women in couples can partly “make up” for the gender wealth gap through being native-born. This is in line with native partners having more bargaining power. Within native couples, the stylized fact that wealthier households have a larger gender wealth gap may point us to a different version of the bargaining power hypothesis: the richest households in the data have a high gender wealth gap. That is, the man in the couple owns more wealth when there is a lot of wealth that could potentially be shared.

## Multivariate Results

We now turn to a multivariate analysis of the relationship between socio-demographic characteristics and the intra-household gender wealth gap in Austria. The outcome variable of interest is the gender-specific wealth gap within the couple; the control variables are all couple- and household-specific. The value added of the multivariate analysis is that it allows us to assess the relationship between our socio-demographic characteristics of interest and the gender wealth gap, while holding all other characteristics constant across households. Thus, we are able to disentangle the role of any one variable from the other socio-demographic characteristics as well as a battery of other couple-level controls.

Given the focus of the analysis on socio-demographics, the specifications in Tables 5, 6, 7 and 8 have age, education, fertility, marital status, and immigration^9^ as their main explanatory variables. Along with the socio-demographic characteristics, the models include what we call “labor controls” and “wealth controls.” The former include indicators of the labor market situation in the couple: mutually exclusive categories of whether the man only, the woman only, neither or both partners are employers, employees, unemployed, self-employed, or not in the labor force, as well as an indicator of the difference in the work histories (number of years worked) of the man and the woman. The wealth controls are a dummy variable indicating that the household has received an inheritance or gift and the inverse hyperbolic sine transformed level of wealth owned by the couple. For reasons of space, these are not displayed in detail in this section; see Table 12 in the Appendix for the coefficients of the full set of control variables corresponding to the estimations in Table 5.

Table 5 presents the baseline results. The models (1)–(4) add the socio-demographic controls sequentially (while all models control for the labor and wealth controls). The first model controls for age differences. The second model adds controls for migratory background. The third model adds the other socio-demographic controls, and finally, the fourth model adds the control for the gender of the respondent.

Across these model specifications, two of our socio-demographic characteristics of interest are consistently highly statistically significant predictors of a gender wealth gap within a couple. First, couples in which the woman—and only the woman—is an immigrant have a higher gender wealth gap. Second, couples in which the respondent is female have a lower gender wealth gap. Other variables which have some effect, depending on the specification, are a large age gap favoring the older partner. That is, when the man is more than ten years older than the woman, then the gender wealth gap is larger in model (1), and when the woman is more than ten years older, then the gender wealth gap is reduced in models (2) and (3). Furthermore, the highest education in couples is negatively correlated with the gender wealth gap in the full specification (4). The additional controls for labor market and wealth effects are mostly statistically insignificant. However, the gender wealth gap is higher when the woman’s labor market attachment is weak (not in the labor force or unemployed), and when the household inherited (as seen in Table 12 in the Appendix).

**Table 5:** OLS results: socio-demographic determinants of the intra-household gender wealth gap

	(1)	(2)	(3)	(4)
Man older, $\Delta<$ 5	0.684	0.654	0.685	0.776
	(0.617)	(0.604)	(0.588)	(0.587)
Man older, 5 $\le \Delta<$ 10	1.213	1.161	1.134	1.235
	(0.764)	(0.772)	(0.764)	(0.770)
Man older, $\Delta \ge$ 10	1.749*	1.468	1.515	1.577
	(1.004)	(1.034)	(0.994)	(0.971)
WoMan older, $\Delta<$ 5	0.903	0.946	0.994	1.118
	(0.740)	(0.734)	(0.712)	(0.718)
Woman older, 5 $\le \Delta<$ 10	− 1.156	− 1.118	− 0.966	− 0.946
	(1.225)	(1.227)	(1.245)	(1.238)
Woman older, $\Delta \ge$ 10	− 3.934*	− 3.916*	− 3.836*	− 3.182
	(2.228)	(2.254)	(2.283)	(2.231)
Avg. age of couple	− 0.007	− 0.008	0.002	0.003
	(0.021)	(0.021)	(0.027)	(0.027)
Female immigrant only		2.375***	2.534***	2.506***
		(0.894)	(0.891)	(0.867)
Male immigrant only		− 0.194	− 0.063	− 0.172
		(0.869)	(0.831)	(0.808)
Both immigrants		1.401	1.265	1.260
		(0.847)	(0.887)	(0.887)
Man more ed., 1 cat.			0.310	0.295
			(0.544)	(0.545)
Man more ed., 2 cats.			0.460	0.409
			(1.377)	(1.360)
Woman more ed., 1 cat.			− 1.104	− 0.850
			(0.796)	(0.826)
Woman more ed., 2 cats.			1.248	1.606
			(4.927)	(4.960)
Highest education in couple			− 0.657	− 0.804*
			(0.422)	(0.419)
One child			− 0.363	− 0.239
			(0.547)	(0.536)
2$+$ children			− 0.187	0.059
			(0.451)	(0.457)
Youngest child 0–5			0.680	0.727
			(0.651)	(0.647)
Married			0.125	0.028
			(1.258)	(1.231)
Married, born before 1958			− 0.254	− 0.112
			(0.734)	(0.729)
Female respondent				− 1.711***
				(0.363)
*N*	1503	1503	1503	1503
${R}^{2}$	0.051	0.060	0.068	0.086

Full sample

This table predicts the socio-demographic determinants of the mean intra-household gender wealth gap in couples (i.e., the IHS transformed difference between the male’s and the female’s net wealth). $\Delta$ indicates the difference between the man’s and the woman’s variable value. The variables included in the wealth and labor market controls are described in the text. Standard errors in parentheses

*$p<0.1$, **$p<0.05$, ***$p<0.01$. Authors’ calculations on 2014 HFCS data

About three quarters of couples in the sample claim to share their wealth equally. In other words, most households report having no gender wealth gap at all. In our next model, we therefore ask how our socio-demographic variables are related to the gender wealth gap in households with an unequal distribution of wealth. Table 6 presents the results. The story that emerges is largely the same as in the baseline results, with some slight differences.

In the couples that report an unequal distribution of wealth, the strongest and most consistent predictors of the size of the gender wealth gap are again whether only the woman is an immigrant (positive), and whether the respondent is female (negative). Furthermore, the age difference within the couple is positive and weakly significant for both when the man is older *and* when the woman is older. Overall, the multivariate analysis shows robustly that in our data the gender wealth gap within couples in Austria is mainly driven by the gender of the respondent, and that households with a native-born Austrian man and a foreign-born female are those with the highest gender wealth gap, on average.

**Table 6:** OLS results: socio-demographic determinants of the intra-household gender wealth gap: only households with a wealth gap

	(1)	(2)	(3)	(4)
Man older, $\Delta<$ 5	3.760	3.629	3.043	3.829
	(3.009)	(2.934)	(2.756)	(2.542)
Man older, 5 $\le \Delta<$ 10	4.589	4.591	4.013	4.909*
	(3.345)	(3.323)	(3.105)	(2.979)
Man older, $\Delta \ge$ 10	5.817	5.260	5.339	5.611
	(3.913)	(4.043)	(3.888)	(3.560)
Woman older, $\Delta<$ 5	3.566	3.846	3.533	4.849*
	(3.277)	(3.176)	(2.896)	(2.843)
Woman older, 5 $\le \Delta<$ 10	− 1.677	− 1.706	− 1.637	− 1.174
	(4.655)	(4.747)	(4.530)	(4.401)
Woman older, $\Delta \ge$ 10	− 5.219	− 5.220	− 4.779	− 2.363
	(4.596)	(4.546)	(5.135)	(4.251)
Avg. age of couple	0.062	0.048	0.058	0.059
	(0.062)	(0.060)	(0.070)	(0.066)
Female immigrant only		4.350**	4.302***	3.454**
		(1.717)	(1.626)	(1.601)
Male immigrant only		− 1.104	− 0.967	− 1.863
		(3.122)	(2.873)	(2.668)
Both immigrants		2.826	2.216	2.000
		(2.142)	(2.307)	(2.307)
Man more ed., 1 cat.			0.285	− 0.018
			(1.714)	(1.626)
Man more ed., 2 cats.			3.491	2.494
			(6.101)	(6.608)
Woman more ed., 1 cat.			− 3.822	− 3.700
			(2.341)	(2.377)
Woman more ed., 2 cats.			0.071	0.481
			(7.700)	(7.982)
Highest education in couple			− 1.850	− 1.863
			(1.426)	(1.396)
One child			− 0.412	− 0.359
			(1.653)	(1.553)
2$+$ children			0.437	1.634
			(1.491)	(1.478)
Youngest child 0–5			2.190	2.844
			(1.935)	(2.005)
Married			1.047	0.463
			(2.155)	(2.109)
Married, born before 1958			− 0.190	0.703
			(2.341)	(2.238)
Female respondent				− 5.740***
				(1.189)
*N*	436	436	436	436
${R}^{2}$	0.140	0.154	0.182	0.238

This table predicts the socio-demographic determinants of the mean intra-household gender wealth gap in couples, where the gap is the IHS transformed difference between the male’s and the female’s net wealth. The subsample comprises only those households who indicated an unequal distribution within the couple. $\Delta$ indicates the difference between the man’s and the woman’s variable value. The variables included in the wealth and labor market controls are described in the text. Standard errors in parentheses

*$p<0.1$, **$p<0.05$, ***$p<0.01$. Authors’ calculations on 2014 HFCS data

The composition of the couple’s migratory background proves to be a very strong indicator of the gender wealth gap. This is an important result, and it raises the question about why this may be the case. As discussed in Sect. 2, there is discrimination against immigrants in Austria and immigrant women are crowded into low-wage jobs, when they are active on the labor market. However, it is impossible to more fully explore the mechanisms behind these results in the HFCS data. In particular, the data indicate only whether a person was born in another country—not the specific country from which they come or their economic conditions upon immigrating. We therefore cannot say anything about cultural norms or economic conditions of the immigrants in our sample that might be driving the results in Tables 5 and 6.

Immigration is a complex topic. There are very different selection mechanisms that influence the choice to immigrate: it is both some of the poorest as well as the richest households who must or can migrate. Moreover, the institutional contexts, inheritance regimes, and possibility for return migration differ greatly by country of origin and economic status. For these reasons, we next consider determinants of the gender wealth gap in the sample of households that have nobody with a migratory background. That is, we drop the approximately 10% of the sample with one or more immigrant in the couple, and we run our analysis with the reduced sample.

Table 7 presents the results of the analysis for the couples in which both partners are native Austrians. The female respondent remains statistically significantly negatively related to the gender wealth gap within the couple. The other striking result, which remains consistent across model specifications is that the strongest driver of the gender wealth gap within couples is now the age difference when the man is more than ten years older.

**Table 7:** OLS results: socio-demographic determinants of the intra-household gender wealth gap

	(1)	(2)	(3)
Man older, $\Delta<$ 5	0.457	0.433	0.502
	(0.567)	(0.551)	(0.550)
Man older, 5 $\le \Delta<$ 10	0.873	0.859	1.006
	(0.768)	(0.765)	(0.776)
Man older, $\Delta \ge$ 10	2.095**	2.196**	2.242**
	(1.062)	(1.076)	(1.076)
Woman older, $\Delta<$ 5	0.885	0.872	0.967
	(0.724)	(0.712)	(0.713)
Woman older, 5 $\le \Delta<$ 10	− 1.888	− 1.759	− 1.737
	(1.256)	(1.254)	(1.260)
Woman older, $\Delta \ge$ 10	− 4.163	− 4.152	− 3.598
	(2.876)	(2.781)	(2.693)
Avg. age of couple	− 0.010	0.007	0.004
	(0.021)	(0.029)	(0.028)
Man more ed., 1 cat.		0.396	0.397
		(0.591)	(0.591)
Man more ed., 2 cats.		− 0.714	− 0.820
		(1.443)	(1.446)
Woman more ed., 1 cat.		− 1.288	− 1.090
		(0.952)	(0.980)
Woman more ed., 2 cats.		− 0.905	0.255
		(0.811)	(0.840)
Highest education in couple		− 0.237	− 0.391
		(0.466)	(0.458)
One child		− 0.205	− 0.035
		(0.599)	(0.591)
2$+$ children		− 0.122	0.099
		(0.533)	(0.539)
Youngest child 0–5		0.828	0.799
		(0.834)	(0.828)
Married		0.138	0.110
		(1.492)	(1.473)
Married, born before 1958		− 0.481	− 0.335
		(0.763)	(0.769)
Female respondent			− 1.629***
			(0.391)
*N*	1253	1253	1253
${R}^{2}$	.054	.062	.078

Only households with native-born members of the couple

This table predicts the socio-demographic determinants of the mean intra-household gender wealth gap in couples, where the gap is the IHS transformed difference between the male’s and the female’s net wealth. The sample comprises only households in which both members of the couple are native-born Austrians. $\Delta$ indicates the difference between the man’s and the woman’s variable value. The variables included in the wealth and labor market controls are described in the text. Standard errors in parentheses

*$p<0.1$, **$p<0.05$, ***$p<0.01$. Authors’ calculations on 2014 HFCS data

We show the results for the subsample of households in which both members of the couple are native-born Austrians and in which the couple reports having an unequal distribution of wealth (Table 8). Despite the relatively small size of this subsample of couples (just 338 of the original 1,503 meet these criterion), the main findings from the other subsamples studied remain robust. In particular, the gender wealth gap is positively related to a large age difference favoring the man in the couple, and negatively related to the woman being the survey respondent. In addition, when the youngest child is small (age zero to five), then the gender wealth gap is larger.

**Table 8:** OLS results: socio-demographic determinants of the intra-household gender wealth gap

	(1)	(2)	(3)
Man older, $\Delta<$ 5	3.348	2.514	3.536
	(2.978)	(2.937)	(2.862)
Man older, 5 $\le \Delta<$ 10	3.724	2.952	4.474
	(3.280)	(3.252)	(3.307)
Man older, $\Delta \ge$ 10	7.202*	7.319*	7.662*
	(3.928)	(4.001)	(3.929)
Woman older, $\Delta<$ 5	3.836	2.503	4.341
	(3.420)	(3.306)	(3.323)
Woman older, 5 $\le \Delta<$ 10	-4.771	-4.709	-3.961
	(5.647)	(5.560)	(5.439)
Woman older, $\Delta \ge$ 10	-4.871	-5.504	-2.695
	(5.098)	(6.122)	(5.296)
Avg. Age of Couple	0.038	0.078	0.046
	(0.065)	(0.087)	(0.080)
Man more ed., 1 cat.		0.612	0.319
		(1.972)	(1.903)
Man more ed., 2 cats.		0.859	-1.480
		(8.192)	(9.041)
Woman more ed., 1 cat.		-4.301	-4.120
		(2.879)	(2.933)
Woman more ed., 2 cats.		0.000	0.000
		(.)	(.)
Highest education in couple		-1.433	-1.719
		(1.592)	(1.558)
One child		-0.057	0.212
		(1.904)	(1.821)
2$+$ children		-0.528	1.103
		(1.865)	(2.015)
Youngest child 0–5		4.534*	4.509*
		(2.625)	(2.572)
Married		0.503	0.358
		(2.695)	(2.602)
Married, born before 1958		-0.628	0.556
		(3.096)	(2.999)
Female respondent			-6.119***
			(1.376)
*N*	338	338	338
${R}^{2}$	.158	.189	.248

Only couples without an immigrant and those reporting intra-couple wealth inequality

This table predicts the socio-demographic determinants of the mean intra-household gender wealth gap in couples, where the gap is the IHS transformed difference between the male’s and the female’s net wealth. The sample comprises only households in which both members of the couple are native-born Austrians, and among them, only those households who indicated an unequal distribution within the couple. $\Delta$ indicates the difference between the man’s and the woman’s variable value. The variables included in the wealth and labor market controls are described in the text. Standard errors in parentheses

*$p<0.1$, **$p<0.05$, ***$p<0.01$. Authors’ calculations on 2014 HFCS data

Across the board, regardless of subpopulation studied and consistent across models including different covariates,^10^ two interrelated and consistent findings emerge. First, our findings in the multivariate analysis support the hypothesis that socio-demographic variables interact with gender in such a way that bargaining power plays an important role in the couple-level gender wealth gap in Austria. In the full sample including both native and non-native partners, it is the weaker bargaining position of immigrant women who are living with an Austrian man that is the most resounding finding. This is the case irrespective of whether all couples or only those reporting a gender wealth gap are investigated. When the sample is limited to couples in which both partners are natives, then the bargaining power of men who are considerably older than their partners comes to bear. Moreover, the full specification for the full sample shows that the highest level of education within the couple matters: more educated couples have a lower gender wealth gap. This finding could reflect norms of gender equality that may be present in higher education settings, but it could also emerge because of strong patterns of assortative mating in Austria (Augustin et al., 2015). Highly educated men are likely partnered with highly educated women, and formally educated women will be most likely to demand economic equality within the couple. Overall, although the methods applied here do not permit disentangling direct effects of these variables on the gender wealth gap, our findings in the multivariate analysis thus support the hypothesis that bargaining power plays an important role for the couple-level gender wealth gap in Austria.

The second main finding is that couples in which the woman is considered the “financially most knowledgeable person”—proxied by their participation as the respondent to the survey—have considerably smaller gender wealth gaps. This finding first appeared in the bivariate analysis in Table 3 and it has proven robust to the subpopulation and covariate specifications in the multivariate analysis in this section. As with the immigrant and age gap results, the female respondent as an explanatory variable is significant despite numerous other socio-demographic explanatory variables, and a battery of controls that include labor market characteristics and wealth-related controls. Since financial knowledge may indicate higher bargaining power, this supports our first finding that socio-demographic characteristics related to bargaining power play an important role in the gender wealth gap.

## Discussion and Conclusion

One key dimension of gender inequality is the unequal distribution of wealth between men and women. This topic is still under-explored in the literature, and this paper contributes to the discussion by considering how age, education, marital status, fertility, immigration status, as well as the gender of the survey respondent are related to the intra-household gender wealth gap in Austria. A key take-away is that—beyond simple direct effects of these characteristics—bargaining power plays an important role for the couple-level gender wealth gap.

We find a mean net wealth gap of €58,400 among Austrian couples in 2014, corresponding to a gap of 28% of the men’s average wealth; these results are in line with previous research from Italy, France, and Germany. D’Alessio (2018) reconstructs individual wealth data from Italy in 2016 to show that men on average have 25% higher net wealth than women. The observed wealth gaps are attributable to gender differences in age, education, employment, and income. Frémeaux and Leturcq (2020) show an increased individualization of wealth within French couples over time, which is driven by changes in the socio-demographic composition of couples concerning their marital history, since later marriage and divorce become more common. They find that the gender wealth gap is higher for unmarried couples and married couples with a separate property regime—confirming our finding of wealth gaps for both married and unmarried couples. Grabka et al. (2015) show that the German intra-partnership wealth gap is greatest when the man takes financial decisions, and lowest when the woman has the last say, which are the same dynamics that we observe with the gender of the “financially most knowledgeable person.” The authors relate specific characteristics such as self-employment, no migratory background, inheritances, and high income to a decrease in the wealth gap for women, but an increase for men.

We show in bivariate analysis that wealth rises for both men and women with age and education, whereas a migratory background is negatively correlated with wealth. However, the gender-specific wealth gap persists beyond the mitigating factors of age and education: women need to be considerably older for the gender wealth gap to become negative, and the average wealth gap persists even when women are more educated than men. In contrast, being native-born appears to enable women to “catch up” to men’s wealth holdings. Furthermore, we find some descriptive evidence that the gender wealth gap is positively correlated with the level of wealth owned by the household: the more wealth there is, the more likely it is that we observe a larger gender wealth gap. This is the case for married couples, whose gender wealth gap is right-skewed across net wealth.

Multivariate analysis confirms the central role of bargaining power. In the full sample including couples with native and non-native partners, the most resoundingly significant socio-demographic factor explaining the gender wealth gap is a gendered migratory background: the gender wealth gap is particularly high in couples in which the man is native-born and the woman is an immigrant. Limiting the sample to native couples brings out the age difference (in particular when the man is older) as an important variable. Additionally, in couples in which the woman is the “financially most knowledgeable person,” the gender wealth gap is considerably smaller. While this finding also supports the hypothesis that differences in bargaining power correspond to an intra-couple wealth gap, we cannot fully rule out cognitive bias, or gender differences in perceiving, or acting upon, social desirability when responding to the survey.

Our results confirm our initial hypothesis that higher bargaining power for women results in lower intra-couple wealth gaps. Yet, we could not show that a woman’s stronger bargaining position is able to reverse the gap. Socio-demographic characteristics act as proxies for bargaining power, but their relationship with wealth holdings are stronger for men than for women. Although we can proxy bargaining power with the socio-demographic characteristics available in the HFCS, we have no information about the black box of the household’s internal dynamics—how negotiations are carried out or how financial decisions are made.

There is a correlation between certain socio-demographic characteristics that the literature has shown to be associated with higher wealth levels and *men’s* wealth levels, which can be explained via stronger bargaining power. However, we cannot explain with our methodology why we do not find the same degree of correlations between higher bargaining power and women’s higher wealth levels. This suggests that socio-demographic characteristics do not automatically confer women higher bargaining power. While we thus cannot resolve the question whether differences in bargaining power are a cause or a consequence of the intra-household gender wealth gap, our results indicate that a stronger bargaining position of women, such as (substantially) higher age or more financial knowledge, as well as—to some degree—higher education, is associated with smaller intra-household wealth gaps. Finally, an important limitation of our data structure is that we only have information on gaps in aggregated net wealth, and cannot differentiate between wealth gaps in debt, real or financial assets.

Gender wealth inequality matters for many aspects of life and affects other types of inequalities beyond socio-economic ones. Gender bias in health, medical care, architecture, safety, or urban mobility (as discussed by Criado Perez (2019)) all link back to wealth inequalities and are also influenced by bargaining power. Addressing gender wealth inequalities should therefore be a priority for public policy. One option for doing so is for the welfare state to reduce the need to accumulate private wealth, for instance, through statutory pensions (Cordova et al., 2022).

Our findings imply that policy should aim at strengthening women’s bargaining power and wealth accumulation processes. This requires addressing first and foremost gender inequalities in pay, employment, and working hours, since income inequality eventually translates into wealth inequality. This appears to be particularly relevant for immigrant women, whose income earning capacity, bargaining power, and wealth position may be strengthened through institutional arrangements such as easier access to labor markets, residence permits, and citizenship. Beyond labor market measures, our results suggest that investing in women’s education and financial literacy would reduce gender wealth gaps, especially since higher education may translate to more financial literacy and thus higher wealth levels (Cupák et al., 2018).

Since this is the first investigation of socio-demographic explanations of the gender wealth gap within households in Austria, many research questions remain open. First and foremost, our results beg the question of whether the relevance of intra-couple bargaining power over wealth apply to other countries. Second, a more in-depth analysis of the exact conditions of immigration and the disadvantages facing immigrant women in Austria would help to explain the strong results regarding immigration and the gender wealth gap. Third, other data could potentially move past a major question mark in this study: the gender wealth gap as measured in our data depends on people and households acknowledging to an interviewer that their household resources are held unequally. Register data and in-depth qualitative interviews could potentially help provide more information about the existence and extent of intra-household wealth inequality, as well as the gender differences in reporting it.

## Appendix

See Figs. 2, 3 and 4 and Tables 9, 10, 11, 12, 13.

**Table 9:** Intra-household wealth gap, by gender of respondent and couple marital status

	N	Share with no gap	Mean gap—all	Gap—when any
*Female respondents*	700	77.5	− 2.8	− 12.0
Married	653	79.6	− 1.6	− 8.6
Cohabiting	26	65.3	− 5.2	15.7
Other relationship	21	38.6	− 29.4	− 55.8
Children present	241	79.7	1.1	4.8
No children present	459	76.2	− 4.9	− 20.0
*Male respondents*	803	77.3	8.5	29.3
Married	746	79.2	7.3	28.9
Cohabiting	31	56.5	18.0	18.8
Other relationship	26	43.2	34.8	42.9
Children present	201	76.1	8.8	34.1
No children present	602	77.6	8.4	27.6

This table shows the mean gender wealth gap, relative to male wealth, by the gender of the respondent and the family composition (marital status and children) of the couple. “Married” means that both partners are married; “cohabiting” means that both members of the couple are legally single; and “other” any other combination of partners who are married, legally single, legally partnered but not married, divorced, or widowed. “Has children outside HH” refers to respondents with children living outside of the current household. “Mean gap—all” is the average gap for all households in the sub-population; “mean gap—when any” is the mean gap conditional on the households reporting an uneven wealth ownership. Authors’ calculations on 2014 HFCS data

**Table 10:** Descriptive statistics on labor and wealth controls

	Females	Males
Share employee	47.9	51.6
Share employer	1.9	4.9
Share self-employed	3.7	4.3
Share unemployed	2.0	1.9
Share not in LF	17.1	1.1
Share retired	27.5	36.1
Part-time share	45.2	5.7
Full-time share	54.8	94.3
Work attachment history	0.70	0.88
Years worked	21.2	29.7
Share with employee income	52.9	55.0
Average value employee income	21,440	35,473
Share with self-employment income	7.4	10.8
Average value self-employment income	16,829	39,626
Share with other income	31.2	39.5
Average value other income	13,368	23,411
Total income	16,757	33,029
Share of households with inheritance	31.2	
N observations	1503	1503

Authors’ calculations on 2014 HFCS data

**Table 11:** Wealth and wealth gaps by detailed socio-economic characteristics

	Sample	Share	Mean	Mean gap	Median	Median gap
*Same age (* $\Delta$ * = 0)*						
Women	132	8.6	190,777	− 0.2	107,941	6.5
Men			190,455		115,442	
*Woman is younger*						
$\Delta < 5$ years						
Women	699	45.3	159,205	7.3	68,833	12.7
Men			171,657		78,890	
$5 \le \Delta < 10$ years						
Women	311	20.7	137,614	19.2	68,057	22.0
Men			170,411		87,251	
$\Delta \ge 10$ years						
Women	103	7.3	90,448	28.3	38,976	44.3
Men			126,070		69,920	
*Woman is older*						
$\Delta < 5$ years						
Women	192	13.2	141,220	70.8	68,973	16.6
Men			483,521		82,658	
$5 \le \Delta < 10$ years						
Women	48	3.5	115,134	− 16.6	79,877	− 14.7
Men			98,764		69,632	
$\Delta \ge 10$ years						
Women	18	1.4	199,328	− 97.0	173,721	− 438.9
Men			101,198		32,239	
*Same education (*$\Delta$ *= 0)*						
Women	964	63.7	140,813	36.2	68,219	13.7
Men			220,740		79,069	
*Woman less educated*						
$\Delta$ = 1 category						
Women	380	25.5	165,168	10.7	61,391	29.0
Men			184,886		86,513	
$\Delta$ = 2 categories						
Women	26	1.7	97,483	21.7	68,419	26.2
Men			124,554		92,702	
*Woman more educated*						
$\Delta$ = 1 category						
Women	127	8.8	162,779	11.9	94,017	− 7.0
Men			184,830		87,840	
$\Delta$ = 2 categories						
Women	6	0.4	351,922	7.4	168,288	− 11.3
Men			380,191		151,200	
*Married*						
Women	1,399	92.8	154,034	28.8	73,661	15.6
Men			216,343		87,290	
*Unmarried*						
Women	104	7.2	84,792	8.7	21,010	30.0
Men			92,833		30,030	
*No children*						
Women	873	56.0	136,591	39.5	65,752	13.7
Men			225,720		76,186	
*One child*						
Women	253	17.0	150,706	0.7	65,520	− 1.2
Men			151,799		64,769	
*Two children*						
Women	276	18.9	149,314	18.1	92,058	25.9
Men			182,273		124,305	
*Three+ children*						
Women	101	8.1	231,832	9.8	58,624	1.4
Men			257,146		59,448	
*Youngest child 0–5*						
Women	199	13.7	79,801	25.9	22,732	42.4
Men			107,751		39,475	
*Youngest child 6–12*						
Women	174	11.7	178,606	9.6	68,863	− 1.4
Men			197,610		67,888	
*Youngest child 13–17*						
Women	104	7.2	157,623	7.2	106,696	− 0.3
Men			169,867		106,423	
*Youngest child 18+*						
Women	153	11.4	258,160	5.0	161,100	5.3
Men			271,652		170,071	
*Neither immigrant*						
Women	1,253	84.2	152,013	30.9	84,364	12.8
Men			219,981		96,793	
*Both immigrant*						
Women	102	6.5	53,961	− 3.2	9,770	− 9.3
Men			52,305		8,940	
*Only female immigrant*						
Women	75	4.8	102,255	23.3	51,106	40.7
Men			133,376		86,204	
*Only male immigrant*						
Women	73	4.5	280,321	− 1.4	46,152	29.0
Men			276,417		64,963	

Gender wealth gap relative to male wealth. Authors’ calculations on 2014 HFCS data. Survey weights and multiple imputation structure employed in all calculations

**Table 12:** OLS results: labor and wealth controls (corresponding to Table 5)

	(1)	(2)	(3)	(4)
Work history diff.	0.097	0.057	0.057	0.052
	(0.585)	(0.576)	(0.580)	(0.585)
Employer^f^ only	0.346	− 0.035	0.396	0.394
	(1.674)	(1.690)	(1.725)	(1.733)
Employer^m^ only	1.452	1.399	1.509	1.794
	(1.409)	(1.400)	(1.374)	(1.352)
Both employers	− 4.140	− 3.695	− 3.775	− 3.355
	(2.889)	(2.843)	(2.893)	(2.867)
Employee^f^ only	0.176	0.146	0.410	0.231
	(1.116)	(1.121)	(1.108)	(1.093)
Employer^m^ only	− 1.224	− 1.472*	− 1.437*	− 1.107
	(0.778)	(0.777)	(0.776)	(0.779)
Both employees	1.525	1.786	1.758	1.786
	(1.162)	(1.174)	(1.168)	(1.175)
NILF^f^ only	2.001***	2.025***	1.997***	1.869***
	(0.671)	(0.653)	(0.650)	(0.660)
NILF^m^ only	− 4.261	− 4.337	− 4.161	− 3.966
	(3.793)	(3.752)	(3.799)	(3.760)
Both NILF	3.675	3.897	4.547	3.861
	(4.311)	(4.252)	(4.462)	(4.412)
Unemployed^f^ only	1.766	1.813*	1.872*	1.888*
	(1.087)	(1.059)	(1.136)	(1.097)
Unemployed^m^ only	− 1.720	− 1.914	− 1.655	− 1.628
	(1.614)	(1.657)	(1.720)	(1.791)
Both unemployed	− 0.414	− 0.476	− 0.939	− 1.242
	(3.686)	(3.721)	(3.780)	(3.791)
Self-employed^f^ only	1.923	2.152	2.636	2.669
	(1.624)	(1.654)	(1.737)	(1.775)
Self-employer^m^ only	0.422	0.210	0.370	0.790
	(1.311)	(1.363)	(1.397)	(1.401)
Both self-employed	0.243	0.205	− 0.158	− 0.519
	(3.523)	(3.562)	(3.661)	(3.651)
Net wealth level	0.040	0.052	0.065*	0.063
	(0.038)	(0.037)	(0.039)	(0.039)
Household inherited	1.008**	1.022**	1.116**	1.128**
	(0.480)	(0.474)	(0.479)	(0.487)
Constant	− 0.859	− 1.075	− 0.493	0.366
	(1.736)	(1.748)	(2.364)	(2.331)

This table predicts the socio-demographic determinants of the mean intra-household gender wealth gap in couples (i.e., the IHS transformed difference between the male’s and the female’s net wealth). The superscript “*f*”/“*m*” beside the variable name indicates that the variable applies to the female/male partner. Standard errors in parentheses. *$p<0.1$, **$p<0.05$, ***$p<0.01$. Authors’ calculations on 2014 HFCS data

**Table 13:** Quantile regression results: socio-demographic determinants of the intra-household gender wealth gap

	p25	p50	p75
Man older, $\Delta<$ 5	4.833	6.903	0.060
	(4.077)	(6.200)	(1.222)
Man older, 5 $\le \Delta<$ 10	6.742	6.660	0.671
	(4.435)	(6.162)	(1.248)
Man older, $\Delta \ge$ 10	7.704	7.220	1.026
	(4.960)	(6.445)	(1.391)
Woman older, $\Delta<$ 5	5.306	7.987	0.989
	(5.415)	(6.472)	(1.418)
Woman older, 5 $\le \Delta<$ 10	4.459	2.452	− 2.260
	(7.786)	(8.205)	(1.714)
Woman older, $\Delta \ge$ 10	3.308	− 7.173	− 16.681
	(6.384)	(9.935)	(24.901)
Avg. Age of Couple	0.015	0.070	0.060***
	(0.119)	(0.103)	(0.022)
Immigrant^f^ only	8.600	1.090	− 0.322
	(5.699)	(3.707)	(0.590)
Immigrant^m^ only	− 0.349	− 2.557	− 0.657
	(6.351)	(2.871)	(0.772)
Both immigrants	7.295	− 0.248	− 0.814
	(5.408)	(2.177)	(0.593)
Man more ed., 1 cat.	0.229	0.006	− 0.035
	(2.456)	(1.902)	(0.359)
Man more ed., 2 cats.	− 1.960	3.841	0.550
	(12.610)	(14.779)	(3.813)
Woman more ed., 1 cat.	− 2.469	− 10.109	− 0.645
	(4.064)	(6.281)	(0.744)
Woman more ed., 2 cats.	− 12.167	4.910	0.667
	(39.301)	(11.886)	(2.390)
Highest education in couple	− 3.137	− 0.626	0.150
	(2.260)	(1.561)	(0.433)
One Child	− 0.762	− 0.195	− 0.389
	(2.593)	(2.264)	(0.446)
2$+$ Children	1.712	0.798	0.859**
	(3.415)	(2.161)	(0.435)
Youngest child 0–5	1.814	2.684	0.440
	(4.087)	(2.965)	(0.560)
Married	3.023	− 0.631	− 0.781
	(2.961)	(2.978)	(0.625)
Married, born before 1958	− 1.270	0.677	0.321
	(4.054)	(3.024)	(0.668)
Female respondent	− 6.142**	− 5.454*	− 1.373***
	(2.524)	(2.982)	(0.396)
*N*	436	436	436
$R^{2}$	.028	.033	.024

Only households with native-born members of the couple

This table predicts the socio-demographic determinants of the mean intra-household gender wealth gap in couples, where the gap is the IHS transformed difference between the male’s and the female’s net wealth. The sample comprises only households in which both members of the couple are native-born Austrians. $\Delta$ indicates the difference between the man’s and the woman’s variable value. The variables included in the wealth and labor market controls are described in the text. Standard errors in parentheses

*$p<0.1$, **$p<0.05$, ***$p<0.01$. Authors’ calculations on 2014 HFCS data

## Footnotes

1 D’Alessio (2018) estimates the intra-household distribution of wealth for Italy based on the Bank of Italy’s Survey of Household Income and Wealth. However, since individual information is not available for all components of wealth, the author uses reconstructed data and not actual survey data.

2 Since we are concerned with *intra-couple* wealth inequality, we abstract here from period effects regarding differences in macro-economic circumstances, which we surmise are more likely to affect the level of wealth, and not directly the inequality of wealth between partners.

3 Since the data used in the analysis are a cross section from 2014, the average age of the couple captures information about the institutional conditions of the birth cohort of the people in the couple.

4 Or parental wealth, see Charles et al. (2012).

5 The General Civil Code still forms the basis for Austrian civil law, and determined family law until the 1970s.

6 According to a survey conducted in 2013 by the Austrian Chamber of Notaries, this number was 5%.

7 The transformation applied is $NW=asinh(NW)=ln(NW+\root \of {NW^2+1})$.

8 Note again that the number of observations in some of these pairings is too small to interpret confidently.

9 Since Tables 7 and 8 exclude immigrants from our sample, they omit the controls for immigration.

10 Quantile regression results at the 25th, 50th, and 75th percentile confirm the results of the OLS analysis (see Table 13 in the appendix).
